# The Italian version of the mobile phone problematic use scale for adults (MPPUS): A validation study

**DOI:** 10.1016/j.heliyon.2022.e12209

**Published:** 2022-12-10

**Authors:** Mirian Agus, Maria Lidia Mascia, Natale Salvatore Bonfiglio, Maria Pietronilla Penna

**Affiliations:** Department of Pedagogy, Psychology, Philosophy, University of Cagliari, Italy

**Keywords:** Smartphone addiction, Problematic use of mobile phones (PSU), Withdrawal, Craving, Adults, Questionnaire, Psychometric properties

## Abstract

The Mobile Phone Problematic Use Scale (MPPUS) is a self-report measure developed to identify the problematic use of mobile phones (PSU) among adults. The purpose of this study was to create an Italian version of this scale. A sample of 568 Italian adults completed the MPPUS, presented in association with another validated scale for the assessment of smartphone addiction.

We carried out exploratory factor analyses on the MPPUS. Findings emphasised that the Italian version of the MPPUS fits a bi-factor model, in which the general factor ‘PSU’ was found, including two additional specific factors (i.e., ‘Withdrawal and social aspects’ and ‘Craving and escape from other problems’).

The MPPUS was correlated with the Smartphone Addiction Scale short version. With respect to criterion-oriented validity, the MMPUS was also evaluated in relation to socio-demographic variables (i.e., age and gender). The internal consistency and temporal stability of the scales (test–retest assessment after three months) were confirmed.

## 1 Introduction

The smartphone has become an essential tool in everyone's life. Since 1983 (when the first mobile phone appeared), there has been significant growth in its daily use from childhood to older age [1, 2]. This development may be related to the combination of the mobile phone and the Internet. Thanks to this union, the smartphone allows us to perform many functions and to stay in touch with the rest of the world.

The massive use of smartphones in everyday life undeniably has a number of advantages for the individual, but also a number of disadvantages, highlighted in the literature [3, 4, 5]. One point of weakness is its immoderate use, which can take the features of a real addiction [6, 7]. Similar to all dependences, the consequences are linked to problems of adaptation to the environment, which, if not managed properly, lead to pathology [8]. In general, the domains on which smartphone addiction acts are wide and remarkable [9, 10]. A significant fallout is highlighted on the quality of life; indeed, many cognitive processes are affected, such as attention, sleep, self-location, emotional intelligence, and psychomotricity [10, 11, 12, 13]. Furthermore, smartphone addiction is related to problems in time saturation and in general cognitive overload that also affect the development of human relationships [14, 15, 16, 17, 18]. The exponential and increasing smartphone use can lead to severe consequences, specifically in relation to the age groups of users [19, 20], particularly young adolescents [21, 22]. Adults have also shown problems related to excessive smartphone use. Smetaniuk [23], in a sample of 362 employed US adults, highlighted that lower age, depression, and extraversion predicted higher scores on measures of problematic mobile phone use (PSU). Nahas et al. [24] showed that higher PSU levels are stronger in young (18–34 years) and unmarried people.

Other studies focus on gender differences in PSU. For example, in a sample of medical college students, Chen et al. [25] found that playing games on smartphones predicted addiction for male students, whereas using multimedia and social networking applications was a predictor for females. Other scholars pay attention to gender differences in PSU and in Internet application preferences [26]. For example, Lee et al. [27] observed that the smartphone usage for videos, music, or games was strongly related with PSU among males; meanwhile, smartphone usage for social media, chatting, or texting was strongly associated with PSU among females.

In light of these findings, we aimed to perform an Italian validation of the Mobile Phone Problematic Use Scale (MPPUS) [27]. Through the development of the MPPUS, Bianchi and Phillips attempted to find which social and psychological dimensions are strictly tied to behavioural and technological addiction. They focused on the dimensions of tolerance, escape from other problems, withdrawal, craving, and negative life consequences in the areas of social, familial, work, and financial difficulties. The MPPUS was originally designed and validated for adults (aged 18–85 years). Since the first validation, many social aspects and behaviours have changed, and a generational change has occurred in terms of habits and transformations in time management and human relations. There has been a shift from the real to the virtual, which in some cases has led to withdrawal from social life (an example of which is the phenomenon of hikikomori, highlighted by Kato et al. [28]).

PSU has increased over time and varies considerably by country [29]. Around the word, we can find many studies about tools that measure the various dimensions correlated for what constitutes problematic behaviour or risk of addiction. In Italy, some other tools exist to measure PSU and smartphone addiction in relation to different age groups [30, 31, 32, 33, 34]. However, we decided to focus on an instrument that measures additional and complementary dimensions to other instruments validated in Italy. This is due to the need to add dimensions (i.e., the relation between craving and escape from other problems among adults) to be measured in relation to the rampant phenomenon. At the same time, validating a tool that is already used in many countries can facilitate evaluations referred to different populations.

### 1.1 Instrument validation around the world

The MPPUS is a self-report instrument developed by Bianchi and Phillips [27]. It is designed for adult populations to assess PSU features. The content of the 27 items deals with several mobile phone topics: tolerance, escape from other problems, withdrawal, craving, negative life consequences, and negative effects on health, including also control issues over mobile phones and everyday problems due to mobile phone usage. The original version of the questionnaire highlights a single factor related to PSU; the authors reported a Cronbach's alpha of 0.93, indicating high internal consistency. The MPPUS [27], in all its validations, has similarities to and differences from the original version in the samples of administration, the dimensions highlighted, and the final number of items identified for the assessment. Indeed, the adaptation and validation of the MPPUS-27 in different countries underline a variable factor structure and specific factors, as well as the application of different Likert scales (Table 1). The original version uses a 10-point Likert scale with the extremes characterised by 1 (*not at all true*) and 10 (*very true*). Administration in other nations uses different scales of measure [35]; for the Italian version, a five-point Likert scale (from 1 = *not at all true* to 5 = *very true*) is used.

**Table 1:** International validations of the MPPUS.

Study	Sample	Number of Participants	Number of Factors	Item	Scale Used
[36]	Spanish (12–18 y)	1,132 adolescents	Three (dependence, withdrawal, and negative consequences)	MPPUS-26	1–10-point Likert from 1 (not true at all) to 10 (totally true)
[37]	British (11–18 y)	1,026 adolescents	Three (dependence, withdrawal, and negative consequences)	MPPUS-26	1–10-point Likert from 1 (not true at all) to 10 (totally true)
[38]	Turkish (18–30 y)	300 university students	One	MPPUS-27	1–5-point Likert from 1 (not true at all) to 5 (totally true)
[39]	Japanese (18–25 y)	504 university students	One	MPPUS-27	1–10-point Likert from 1 (not true at all) to 10 (totally true)
[40]	Iranian (20–30 y)	600 university students	Three (mobile phone overuse, withdrawal symptoms, and preoccupation)	MPPUS-24	1–5-point Likert from 1 (not true at all) to 5 (totally true)
[35]	German, Swiss (12–17 y) (Health Effects Related to Mobile Phone Use in Adolescents)	412 adolescents	Five (loss of control, withdrawal, negative life consequences, craving, and peer dependence)	MPPUS-10	1–5-point Likert from 1 (not true at all) to 5 (totally true)
[41]	Spanish (16–65 y)	1,126 adolescents and adults	Four (abuse and excessive phone use, loss of control, social context-induced craving, and tolerance)	MPPUS-26	1–10-point Likert from 1 (not true at all) to 10 (totally true)
[24]	Lebanese (18–65 y)	207 adults	Three (dependence, withdrawal, and negative consequences)	MPPUS-10	1–10-point Likert from 1 (not true at all) to 10 (totally true)
[42]	Polish (18–38 y)	640 university students	Five (loss of control, withdrawal, negative life consequences, craving, and peer dependence)	MPPUS-10	1–10-point Likert from 1 (not true at all) to 10 (totally true)

Denoting the possible relevance and applicability of the MPPUS, over and above its present unavailability in Italy, the main aim of this study is to offer data on the factorial structure and psychometric properties of the Italian version of this questionnaire in both explorative and confirmative approaches. We refer to the dimensions identified by Bianchi and Phillips [27] in devising the MMPUS, which studied and included the indicators regarding tolerance, escape from other problems, withdrawal, craving, and negative life consequences. We had to evaluate which dimensions and questions included in the first version of the scale may be useful today to assess PSU in the Italian context. Regarding the confirmative models applied, we compared the fit of four factorial models identified for the MMPUS in other countries to identify the model having the best fit in the Italian context. Our evaluations were conducted by stratifying and analysing the sample according to the socio-demographic variables of gender and age.

## 2 Method

### 2.1 Participants

The participants were 568 Italian adults (421 females – 74.1%) with a mean age of 21.7 (standard deviation [SD] = 6.4, range 18–60 years) (Table 2). The sampling was non-probabilistic. The adults participated voluntarily in the research after completing a consent form. The participants complied with the research protocol by online administration with the Lime survey platform. They were informed about the study through a text briefly describing the features of the research. They were also assured that their responses would remain confidential. Informed consent was obtained from all participants. Ethical guidelines were followed; all procedures performed were in accordance with the ethical standards proposed by national and international organisations (the Italian Association of Psychology, the American Psychological Association, and the 1964 Helsinki Declaration and its succeeding modifications). The study was approved by the Institutional Review Board of the University of Cagliari (n. 6UNICA_DPPF).

**Table 2:** Fit Indices for the confirmatory factorial models assessed.

model	chi^2^	df	P	Ratio chi^2^/df	RMSEA	RMSEA 90% CI	CFI	TLI	WRMR
1) First order CFA – one factor	744.40	252	<.001	2.95	.09	.08–.09	.90	.89	1.33
2) First order CFA – two factors	550.92	251	<.001	2.19	.07	.06–.07	.94	.93	1.10
3) First order ESEM – two factors	466.03	229	<.001	2.03	.06	.05–.07	.95	.94	.94
4) Bi-factor CFA – two factors	438.39	250	<.001	1.75	.05	.04–.06	.96	.95	.87

*Note*. df: degree of freedom; RMSEA: root mean square error of approximation; 90% CI: 90% confidence Interval; CFI: comparative fit index; TLI: Tucker-Lewis index; WRMR: weighted root mean square residual.

### 2.2 Materials and procedure

#### 2.2.1 Mobile phone problematic use scale (MPPUS)

The MPPUS [27] includes 27 items. It is designed for adult populations to assess PSU features. For the Italian version, a five-point Likert scale (1 = *not at all true* to 5 = *very true*) was applied. To develop the Italian version of the questionnaire, it was translated from English to Italian and then back-translated to English, blind to the original version [43].

#### 2.2.2 Smartphone Addiction Scale (SAS)

The participants also completed the SAS-short version, which is validated in Italy [31] to assess the risk level of smartphone addiction [44]. This scale is characterised by items describing daily troubles in life, expectations, withdrawal, relationships on the Internet, abuse, and tolerance (Cronbach's α = 0.79). The items were assessed by a six-point Likert scale (from 1 = *strongly disagree* to 6 = *strongly agree*). The higher scores label individuals with greater levels of smartphone addiction [31]. Regarding our participants, the scale reported a mean of 20.30 (SD = 6.97).

### 2.3 Statistical and data analyses

A multi-stage approach was applied in this study. The participants were randomly divided in two subsamples, in which were carried out different complementary psychometric assessments [45, 46]: the initial stage of the analysis (exploratory factor analysis [EFA]; n = 260) was carried out in the first subsample [47]; the following stage of analysis (confirmatory factor analysis [CFA]; n = 308) was applied for the remaining participants [45, 48].

The adequacy of the subsample sizes for EFA (n = 260) and CFA (n = 308) was assessed with reference to the recommendations provided in the literature [46, 49, 50, 51]. In the literature, the EFA rules of thumb regard the minimum number of participants (e.g., from 100 to 250) [52] and the ratio of participants (*N*) to variables (v), suggested to be 5:1 [49]. Other scholars suggest considering the strength of item loadings, the number of item loadings of each factor, and the communalities [50, 53]; these statistical data have been evaluated to ensure the stability and replicability of EFA solutions [54].

The suitability of subsample size for the application of CFA (n = 308) was also assessed following the literature recommendations [46, 49, 51]. In our study, the following aspects were assessed: the number of relationships among variables, the data scaling (e.g., ordinal), the robust estimator applied (e.g., weighted least square mean and variance [WLSMV] adjusted), and the model complexity [55, 56].

### 2.4 Stage 1

EFA was carried out by applying the principal axis factorilisation method (PAF) [57], with bootstrapping (n = 500) and Promax rotation of axes [58]. PAF uses a reduced correlation matrix, in which the diagonals are characterised by the communalities of observed variables (e.g., identifying how much variance in the observed variable is explained by the factor structure) [57]. The Kaiser–Meyer–Olkin (KMO) measure and Bartlett's test of sphericity were applied to evaluate the suitability of these data for PAF [57].

In the application of EFA, special attention was devoted to the purpose of defining the optimal number of factors; this attention is related to the discrepancies highlighted in the literature in different countries regarding the number of factors retained in the MPPUS (Table 1). Considering the relevance of this decision regarding the Italian validation of the instrument, a series of statistical methods suggested in the literature was applied to identify the optimal number of factors to retain [59]. As suggested by Velicer et al. [60], the following were traditionally evaluated: the scree plot [61], the parallel analysis and quantile of parallel analysis [62, 63], the very simple structure (VSS) complexity [64], and Velicer's minimum average partial (MAP) test [60, 65]. Furthermore, considering the suggestions of recent literature, we also used fit indices to identify the optimal number of factors to retain [59], particularly the root mean square error of approximation (RMSEA) (considering a cut-off of <0.08) [59,66,67] and the standardised root mean square residual (SRMR) (considering a cut-off of <0.04) [67]. Nowadays, the combination of classical methods and fit indices is endorsed by different authors [68, 69]. The optimal number of factors to retain in EFA is then identified considering the directions resulting from the applications of these statistical procedures [59]. The internal consistency of each factor was assessed by the reliability coefficient devised for ordinal data by Zumbo et al. [70, 71].

### 2.5 Stage 2

On the basis of previous EFA results, the second subsample of participants (n = 308) was assessed by the application of CFA [72]. The use of CFA in construct validity research allows to compare some alternative models of relationships among constructs, which often constitute a critical aspect of theory testing [73, 74]. Referring to the recent literature and to previous validations of the MPPUS in different countries, we evaluate this version through the application of four dissimilar confirmative models that compared and considered the potential fit in the Italian context of different factorial structures identified previously by other scholars [74, 75].

The first model was characterised by first-order CFA, testing the model with one factor, coherently with the original factorial structure conceived by the authors of the MPPUS [27]. In this model, all items load on a single general factor.

The second model applied first-order CFA with two factors (independent clusters model of CFA [ICM CFA]), in which each item was allowed to load only with one of two factors, and all cross-loadings were usually constrained to be zero [76]. This model aims to test the fit of the factorial structure highlighted by EFA in the first subsample of our study.

Additionally, a third model with two factors was assessed by the application of exploratory structural equation modelling [73], considered a flexible framework to address complex phenomena. This model joins the CFA and EFA features and overcomes the restrictive ICM CFA assumption (that each item must load on only one factor), allowing items to load with more factors and to correlate in a non-exclusive way with a single factor (as highlighted for the MMPUS in validations carried out in other countries) on the basis of item content and/or substantive theory [73, 76].

A fourth model was assessed by a bi-factor CFA, originally devised by Holzinger and Swineford [77] but recently revalued in the literature, because it shows a more accurate description of abilities than the higher-order models [78]. The bi-factor model assesses the multidimensionality of the instrument considering the potential presence of multiple constructs organised in a hierarchical way [79]. This model assumes a solution with a G general factor and S specific substantive factors, which are estimated when the other loadings are constrained to be zero (in the estimation, the correlations between G and S factors are also constrained to be zero). Then the bi-factor model evaluates if a general global construct (mirrored through the G factor) might occur as a unitary dimension underlying the responses to all items and co-occurs with several more specific factors (S1, S2, … Sn) demarcated by the part of the responses that is unaccomplished by the G factor [79]. This method is applied with great satisfaction regarding the assessment of intelligence [80], quality of life [81], and mood disorders [82].

**Figure 1:**
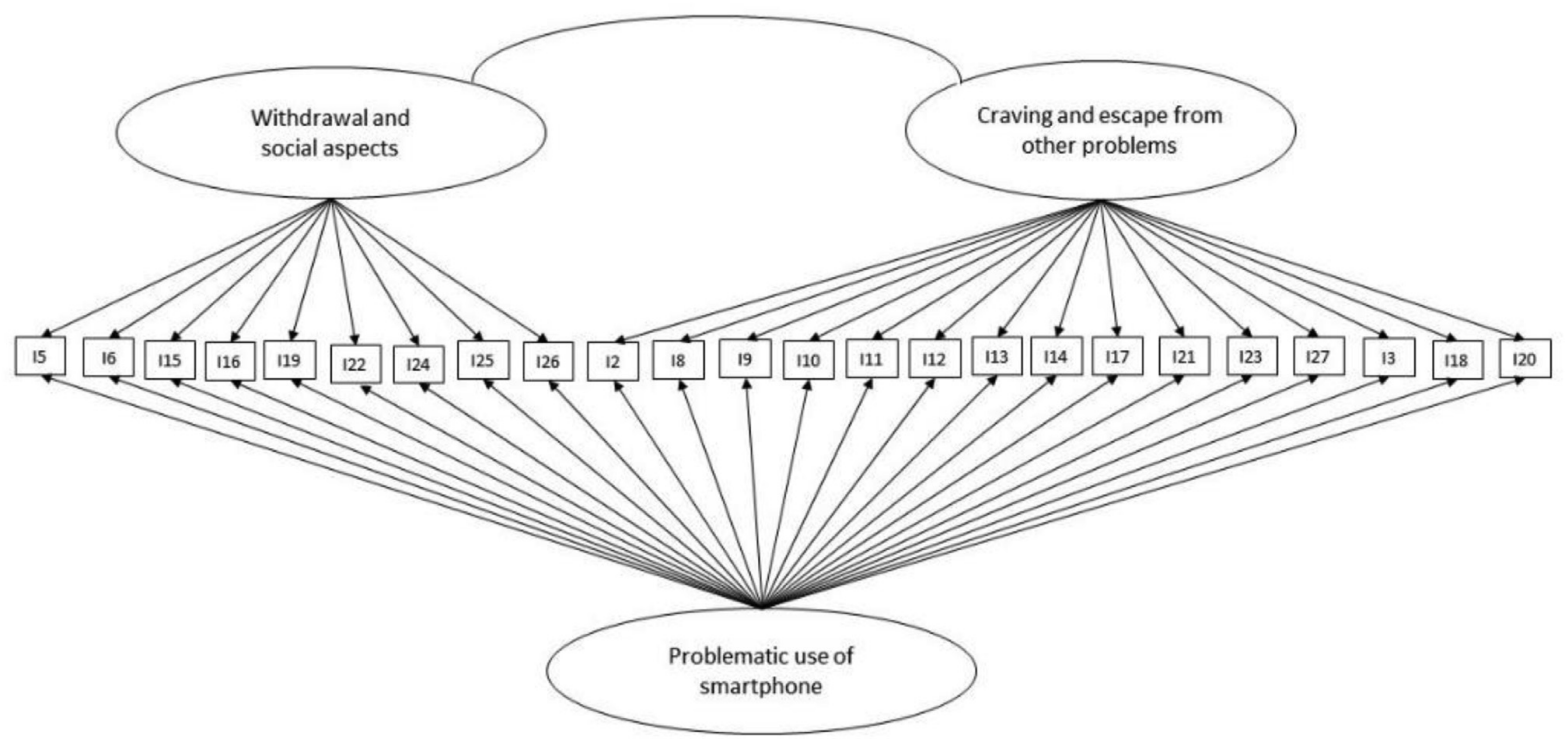
The theoretical bi-factor CFA model tested by model 4.

In our study, this model accounts for the plausibility of a structure with a general dimension of ‘Problematic use of a smartphone’ and, concomitantly, the presence of specific factors related to the dimensions of ‘Withdrawal and social aspects’ and ‘Craving and escape from other problems’ (Figure 1). Regarding the statistical techniques carried out, in confirmative analyses the WLSMV robust estimator was applied (setting the variance of the factors to 1.0) [83,84]. The goodness-of-fit of the models was evaluated on the basis of different indices [85, 86]. The chi-square index is strongly affected by the sample size; then a ratio of Chi^2^ and degrees of freedom (Chi^2^/df) was computed: it is considered acceptable when it is lower than 3 [85]. Also, the comparative fit index and the Tucker–Lewis index (TLI) were reported; their value must be higher than .90 to be considered acceptable [85, 86]. Regarding the application of RMSEA, this index is considered good when it is lower than .05. Furthermore, an RMSEA of .05 and .08 might be considered suitable [85, 86]. Also, the weighted root mean square residual (WRMR) was computed, specifically developed for the application with ordinal data [87]; to designate an adequate fit, their value has to be lower than the .90 cut-off [83].

To evaluate the validity of the questionnaire (criterion and concurrent validity), the scores of the dimensions highlighted in the MPPUS were correlated with the scores of the SAS-SV questionnaire and the variable age. These bivariate relations were assessed applying the parametric correlation Pearson's coefficient index [88]. Furthermore, regarding a criterion-oriented validity, the MPPUS scores were assessed regarding socio-demographic variables (age and gender) by the application of a multivariate analysis of covariance (MANCOVA). Additionally, the temporal stability of the measure was evaluated with test–retest reliability regarding a subsample of participants (n = 99) that fulfilled the protocol after three months. The statistical analyses were carried out with the software M-Plus 7.0 [89] and the software *R* version 4.0 [90]. Specifically, *lavaan*, *semPlot*, *shiny*, and *semTools* packages were applied [91, 92, 93, 94, 95].

## 3 Results

### 3.1 Stage 1

In the first step of the study, the item response distributions and the descriptive statistics were calculated. Then we evaluated the factorial structure of the MPPUS by EFA. To identify the number of factors to retain, the above-mentioned different statistical criteria (scree plot, parallel analysis and quantile of parallel analysis, VSS complexity, Velicer's MAP test, RMSEA, and SRMR) were applied [59, 63, 65]. The screen test, the parallel analysis, the VSS complexity, the Velicer's MAP test suggested that two factors should be retained; the RMSEA and SRMR suggested to retain four factors. Taking these indications and the literature into account, an optimal number of two factors was identified [59, 67, 68]. In EFA, the PAF method was applied, with Promax rotation [58]. The bootstrapping solution (n = 500) was carried out. All items showed high factor loadings in one factor; only three items did not show a clear belonging to one of these factors (items 1, 4, and 7). For this reason, these last items were excluded from the questionnaire. The first factor showed an eigenvalue of 7.82. It comprised items concerning aspects of ‘Withdrawal and social aspects’ (items I5, I6, I15, I16, I19, I22, I24, I25, and I26). The ordinal reliability coefficient for this factor was .87 [70,71]. The second factor had an eigenvalue of 1.27. The items having a high factor loading in this dimension evaluated the aspects regarding ‘Craving and escape from other problems’ (items I8, I9, I2, I3, I10, I11, I12, I13, I14, I17, I18, I20, I21, I23, and I27). The second factor showed a coefficient of ordinal reliability of .91 [70,71]. The two factors showed a Pearson's r correlation of .62.

### 3.2 Stage 2

Accounting for the results of the EFA, in the second subsample, we assessed the factorial structure of the questionnaire by the confirmative approaches (n = 308) [72]. The assessment of these four models allowed, in an efficient way, for an evaluation and comparison of the potential psychometric multidimensionality in the MPPUS. The overall indices related to the application of these four models are reported in Table 2. Model 1 (unidimensional) showed the worst indices of fit; model 4 (bi-factor CFA with two correlated factors) obtained the best indices of goodness-of-fit (Table 2), supporting the appropriateness of the latter model for reproduction of the observed data. The standardised factor loadings for model 4 are shown in Table 3.

**Table 3:** Standardised factor loadings for bifactor CFA model (Model 4) of MPPUS.

Item	F General	F1 **λ**	F2 **λ**
	Problematic use of smartphone	Withdrawal and social aspects	Craving and escape from other problems
5. I have tried to hide from others how much time I spend on my mobile phone	**.66**	**.28**	
6. I lose sleep due to the time I spend on my mobile phone	**.66**	**.26**	
15. I have frequent dreams about the mobile phone	**.31**	**.37**	
16. My friends and family complain about my use of the mobile phone	**.70**	**.63**	
19. I have aches and pains that are associated with my mobile phone use	**.62**	**.32**	
22. I am often late for appointments because I'm engaged on the mobile phone when I shouldn't be	**.67**	**.22**	
24. I have been told that I spend too much time on my mobile phone	**.72**	**.64**	
25. More than once I have been in trouble because my mobile phone has gone off during a meeting, lecture, or in a theatre	**.50**	**.22**	
26. My friends don't like it when my mobile phone is switched off	**.22**	**.18**	
2. I have used my mobile phone to make myself feel better when I was feeling down	**.56**		**.30**
8. When out of range for some time, I become preoccupied with the thought of missing a call	**.47**		**.44**
9. Sometimes, when I am on the mobile phone and I am doing other things, I get carried away with the conversation and I don't pay attention to what I am doing	**.56**		**.17**
10. The time I spend on the mobile phone has increased over the last 12 months	**.64**		**.12**
11. I have used my mobile phone to talk to others when I was feeling isolated	**.44**		**.22**
12. I have attempted to spend less time on my mobile phone but am unable to	**.73**		**.19**
13. I find it difficult to switch off my mobile phone	**.54**		**.48**
14. I feel anxious if I have not checked for messages or switched on my mobile phone for some time	**.51**		**.68**
17. If I didn't have a mobile phone, my friends would find it hard to get in touch with me	**.25**		**.28**
21. There are times when I would rather use the mobile phone than deal with other more pressing issues	**.68**		**.17**
23. I become irritable if I have to switch off my mobile phone for meetings, dinner engagements, or at the movies	**.57**		**.42**
27 I feel lost without my mobile phone	**.51**		**.50**
3. I find myself occupied on my mobile phone when I should be doing other things, and it causes problems	**.82**		.02
18. My productivity has decreased as a direct result of the time I spend on the mobile phone	**.86**		-.04
20. I find myself engaged on the mobile phone for longer periods of time than intended	**.85**		-.01

Note. Bold loadings are significant p < .05.

In model 4 (Figure 1 and Table 3), all loadings related with the general factor were significant (p < .05) and had an acceptable magnitude. Furthermore, three loadings associated with factor 2 were not significant. The general factor corresponds to the dimension originally identified by Bianchi and Phillips [27] regarding the ‘Problematic use of a smartphone’. Factor 1 is connected to ‘Withdrawal and social aspects’, and factor 2 is related to ‘Craving and escape from other problems’. Specifically, regarding our participants, the MPPUS general factor, ‘Problematic use of a smartphone’, has a mean of 1.98 (SD = .580); MPPUS factor 1, ‘Withdrawal and social aspects’, showed a mean of 1.48 (SD = .51); and MPPUS factor 2, ‘Craving and escape from other problems’, presented a mean of 2.26 (SD = .68). As expected on the basis of the literature, these dimensions show high positive bivariate linear correlations with each other, which are statistically significant (p < .001) (Table 4). Indeed, the MPPUS_G has a correlation of r = .82 with MPPUS_F1 and a correlation of r = .94 with MPPSU_F2; MPPUS_F1 and MPPUS_F2 show a correlation of r = .61. Concerning the assessment of the convergent and divergent validities of the MPPUS, we computed Pearson's r bivariate coefficients to evaluate the relations between age, score in the SAS questionnaire, and scores in the MPPUS (Table 4). The findings highlight a modest but significant correlation between age and smartphone addiction for both the SAS and the MPPUS. Furthermore, the correlations between the SAS and the MPPUS are positive and significant for all factors.

**Table 4:** Correlation matrix between age, SAS and MPPUS factors.

		Age	SAS	MPPUS_f1	MPPUS_f2
SAS	Pearson's r	−0.12∗∗			
MPPUS_f1	Pearson's r	−0.09∗	0.58∗∗∗		
MPPUS_f2	Pearson's r	−0.14∗∗	0.76∗∗∗	0.61∗∗∗	
MPPUS_G	Pearson's r	−0.15∗∗∗	0.78∗∗∗	0.82∗∗∗	0.94∗∗∗

Note: ∗p < .05; ∗∗p < .01; ∗∗∗p < .001; MPPUS_G: General Problematic use of smartphone; MPPUS_f1: Withdrawal and social aspects; MPPUS_f2: Craving and escape from other problems.

To control criterion-related validity of the MPPUS, the mean scores on each subscale were evaluated regarding the variable ‘gender’, controlling the effect of the variable ‘age’; a MANCOVA was carried out (definitely, using as between factor the ‘gender’ variable and the ‘age’ as covariate). All assumptions regarding their application were met (Box's M = 6.42, df1 = 3, p = .094; Levene's test for MPPUS_f1 = 3.63, df = 1; 530, p = .057; Levene's test for MPPUS_f2 = .53, df = 1,530, p = .476). The findings highlight a significant effect of ‘age’ and ‘gender’ at the multivariate and univariate levels. The ‘age’ shows a significant but moderate effect, precisely a negative relation with MPPUS scores, characterised by a decrease in the values of problematic use of a smartphone in relation to higher participant age (MPPUS_f1 F = 4.43, df = 1; 529, p = .036, partial eta squared = .008; MPPUS_f2 F = 9.81, df = df = 1; 529, p = .002, partial eta squared = .018).

Furthermore, the variable ‘gender’ has a significant and strong effect for both dimensions (MPPUS_f1 F = 204.85, df = 2; 529, p = .0001, partial eta squared = .436; MPPUS_f2 F = 299.114, df = 2; 529, p = .0001, partial eta squared = .531). Specifically, in the MPPUS_f1, a higher score for males was observed (m_males_ = 1.54, ds_males_ = .56; m_females_ = 1.46, ds_females_ = .50); in the MPPUS_f2, females have a higher score (m_males_ = 2.16, ds_males_ = .64; m_females_ = 2.30, ds_females_ = .69). The test–retest administration to a subsample of participants (n = 99) after three months highlighted Pearson's r correlation coefficients that were positive and significant between the dimensions assessed in two administrations (MPPUS_G r = .46, p < .001; MPPUS_f1 r = .44, p < .001; MPPUS_f2 r = .43, p < .001).

## 4 Discussion and conclusions

This study is devoted to the evaluation of the factor structure and psychometric properties of the Italian version of the MPPUS. The first stage of analysis applied EFA. The EFA highlighted that in the Italian context, only 24 items of the original 27 items devised by the authors might be considered meaningful and useful for assessment with the MPPUS. In relation to the internal structure of this instrument, the second stage of analysis, on the basis of different CFAs applied, suggested that (despite the original unidimensional model devised by the authors) the best solution is a bi-factor model. Indeed, our findings support the idea that the Italian MPPUS identifies a total score of ‘Problematic use of a smartphone’, as well as two additional correlated dimensions, defining ‘Withdrawal and social aspects’ and ‘Craving and escape from other problems’.

Social withdrawal during adolescence and early adulthood is particularly dangerous, as it can lead to serious adaptation problems. In this case, social withdrawal refers to an individual's self-isolation from others and spending excessive time alone using a mobile phone [96]. The other dimension correlated to problematic use of a smartphone is ‘Craving and escape from other problems’ because levels of craving increase following smartphone abstinence, and this aspect can be considered a form of behavioural addiction [97]. The term craving, however, is now also commonly used to indicate a persistent and irresistible desire for a particular substance or conduct with similar effects [98]. In this process of PSU behaviours, associations between craving and the escape motivation are common [99], and loneliness is associated with adolescent PSU [100].

Regarding the psychometric properties of the Italian MPPUS, this version of the questionnaire presented good reliability and validity, coherently with results reported in non-Italian versions of this questionnaire. The intercorrelation between scales was good for all participants, and the three-month temporal stability was good for all MPPUS scores for a subsample of 99 adults. Also, the correlation with the assessment of the SAS-SV questionnaire highlighted significant indices.

The relations among demographic features and the Italian MPPUS scores were also investigated. Concerning the variables of age and gender, our findings showed interesting results, in line with the recent literature about PSU. Specifically, age issues regarding the MPPUS have been investigated in the literature. Some authors evaluated the MPPUS scores regarding different ages, highlighting negative correlations between PSU and age [101]. Indeed, the literature highlights that some age groups may be more at risk of smartphone addiction than others, showing that specific addiction components of smartphone usage were dominant for different age groups, particularly among children and younger adults [20]. For example, youths often use their smartphones even while in bed, before going to sleep, which reduces the quality of sleep [102, 103, 104]. However, our findings highlighted low negative correlations between age and MPPUS scores, specifically for the score of the global factor. These low negative correlations might suggest that the problematic use of smartphones is not only a characteristic of young people but is nowadays a widespread phenomenon for all ages [24, 105, 106, 107, 108, 109, 110, 111, 112]. Indeed, our findings are in line with studies carried out by some authors who, for example, also underlined adults' and parents’ excessive smartphone use [113].

Gender issues about problematic smartphone use are also investigated [114]. Indeed, in the literature the potential role of gender as a predictor of PSU has long been the subject of discussion and attention by psychologists, educators, and researchers [115]. In particular, we sought to compare males and females in order to understand how this socio-demographic variable may influence smartphone overuse in terms of addiction and in relation to the factors that may lead to such overuse. Some studies emphasise that females are more at risk of PSU [37, 116, 117, 118, 119, 120]; fewer studies highlight the opposite [41]. Some studies [42] show no differences between females and males in mobile phone use. This finding is confirmed by Chen et al. [25] in a sample of medical college students. Our findings highlighted that males have significantly higher scores in ‘Withdrawal and social aspects’, but females have the higher score in ‘Craving and escape from other problems’ [26]. These results are consistent with the literature observing problematic smartphone use (e.g., game addiction) by males and use of smartphones by females for social media and social communication [25, 121].

Finally, we must highlight some limitations of this study. The recruitment of participants was non-probabilistic because it was based on the participants’ availability and interest in the topic of the research. Most participants were residents of southern Italian areas, while participants from northern Italian areas were underrepresented. However, we believe that the characteristics that define smartphone use are not related to the geographical area of residence, but to a broader phenomenon that can be defined on a national and global scale [122]. In future studies, it seems important to investigate these dimensions in other populations. Further, this valuation would be more effective if based not exclusively on self-assessment, since self-perception does not always truly report reality; for this reason, it would be interesting to use other non-self-report instruments to add to this scale to get an overall picture of the real presence of an addiction. Very often, individuals are not aware of their excessive use and make an estimate of the time spent on their smartphone [123].

Last, but not least, we highlight the strengths and originality of our study. Considering the widespread use of smartphones, it is of great collective interest to finalise a validated instrument on the Italian adult population, which allows us to detail the dimensions classically identified by the literature on smartphone addiction [27]. Furthermore, considering the worldwide diffusion of the MPPUS, the lack of an Italian version appears to be a flaw in the international context and in the Italian literature about this topic. Additionally, among the strengths of the study is the meticulous evaluation of the factorial structure of the MMPUS by the application of four different confirmative factor models. These models considered the potential fit in the Italian context of the disparate factorial structures highlighted in the validations of the MMPUS in the literature (see Table 1). The fourth model, assessing a bi-factor CFA, showed the best fit, confirming the Italian MMPUS as reliable in a general PSU dimension and in two specific dimensions (‘Withdrawal and social aspects’ and ‘Craving and escape from other problems’). Another strength of this study was the identification of recent potential differences in smartphone addiction in terms of socio-demographic variables, such as age groups and gender.

In conclusion, the MPPUS in this form might be used in educational and research contexts to measure, in an efficient and meaningful way, both general and specific aspects depicting the problematic use of smartphones by adults. In summary, notwithstanding the above-mentioned limitations, results from the present study highlight that the MPPUS is a good measure of problematic use of smartphones in Italian adults. Additionally, the presented results contribute to outlining the potential helpfulness of the total score of the MPPUS as a measure of general PSU in both research and educational contexts.

## Declarations

### Author contribution statement

Mirian Agus: Conceived and designed the experiments; Performed the experiments; Analyzed and interpreted the data; Wrote the paper.

Maria Lidia Mascia: Conceived and designed the experiments; Performed the experiments; Contributed reagents, materials, analysis tools or data; Wrote the paper.

Natale Salvatore Bonfiglio; Maria Pietronilla Penna: Conceived and designed the experiments; Contributed reagents, materials, analysis tools or data; Wrote the paper.

### Funding statement

This research did not receive any specific grant from funding agencies in the public, commercial, or not-for-profit sectors.

### Data availability statement

The data that has been used is confidential.

### Declaration of interest's statement

The authors declare no conflict of interest.

### Additional information

Supplementary content related to this article has been published online at https://doi.org/10.1016/j.heliyon.2022.e12209.

## Appendix A Supplementary data

Appendix A - English version of MPPUS.docx Appendix A - English version of MPPUS.docx

Appendix A - Italian version of MPPUS.docx Appendix A - Italian version of MPPUS.docx
