# Supplementary material for: The Italian version of the mobile phone problematic use scale for adults (MPPUS): A validation study
Source: Heliyon. 2022 Dec 10;8(12):e12209. doi: 10.1016/j.heliyon.2022.e12209 (PMC9764196; doi:10.1016/j.heliyon.2022.e12209)
Supplement: Appendix A - English version of MPPUS.docx [file mmc1.docx]

**Appendix A – Italian version of MPPUS**

| Numbering of items in the original scale | Numbering of items in the Italian version of the scale | Factor in the Italian version | Items in the Italian version |
| --- | --- | --- | --- |
| 2 | 1 | F2 | Ho usato il cellulare per sentirmi meglio quando ero giù |
| 3 | 2 |  | Mi ritrovo a perdere tempo sul cellulare anche quando dovrei stare ad occuparmi di altre cose, e questo è causa di problemi |
| 5 | 3 | F1 | Ho provato a nascondere agli altri quanto tempo effettivamente passo al cellulare |
| 6 | 4 | F1 | Ho sonno arretrato a causa del tempo passato al cellulare |
| 8 | 5 | F2 | Se non ho il telefono sott’occhio per un po’, mi preoccupo per l’eventualità di non sentire qualche telefonata |
| 9 | 6 | F2 | A volte capita che se sono al telefono mentre sto facendo altro mi faccio prendere dalla conversazione e non presto attenzione a ciò che sta succedendo |
| 10 | 7 | F2 | Il tempo che passo al cellulare è aumentato negli ultimi 12 mesi |
| 11 | 8 | F2 | Ho usato il cellulare per parlare con qualcuno quando mi sentivo isolato |
| 12 | 9 | F2 | Ho provato a passare meno tempo al telefono ma non ci sono mai riuscito/a |
| 13 | 10 | F2 | Mi riesce difficile spegnere il cellulare |
| 14 | 11 | F2 | Ho l’ansia se non controllo i messaggi per un po’ o se il mio cellulare, anche solo per brevi periodi, non è acceso |
| 15 | 12 | F1 | Sogno spesso il mio cellulare |
| 16 | 13 | F1 | I miei amici e la mia famiglia si lamentano spesso dell’uso che faccio del cellulare |
| 17 | 14 | F2 | Se non ho il cellulare con me, per i miei amici sarà difficile rintracciarmi |
| 18 | 15 |  | La mia produttività è diminuita in conseguenza al tempo passato al cellulare |
| 19 | 16 | F1 | Ho malesseri e dolori causati dall’uso del cellulare |
| 20 | 17 |  | Mi ritrovo a passare più tempo del previsto al cellulare |
| 21 | 18 | F2 | Ci sono volte in cui preferirei di gran lunga starmene al cellulare che dover avere a che fare con questioni più pressanti |
| 22 | 19 | F1 | Arrivo spesso tardi agli appuntamenti perché mi trattengo al cellulare più del dovuto |
| 23 | 20 | F2 | Divento irascibile se devo spegnere il cellulare per incontri o conferenze, impegni a cena o al cinema |
| 24 | 21 | F1 | Mi è stato detto che spendo troppo tempo al cellulare |
| 25 | 22 | F1 | Più di una volta ho fatto figuracce perché il cellulare ha cominciato a suonare durante incontri, lezioni o in teatro |
| 26 | 23 | F1 | I miei amici non sono contenti se il mio telefono è spento |
| 27 | 24 | F2 | Senza il mio cellulare mi sento perso |

*Note: Factor 1: “Withdrawal and social aspects”; Factor 2: “Craving and escape from other problems”*
